# Interaction between Perceived Action and Music Sequences in the Left Prefrontal Area

**DOI:** 10.3389/fnhum.2016.00656

**Published:** 2016-12-27

**Authors:** Masumi Wakita

**Affiliations:** Department of Neuroscience, Primate Research Institute, Kyoto UniversityInuyama, Japan

**Keywords:** left prefrontal area, sequences, action, music, near-infrared spectroscopy

## Abstract

Observing another person's piano play and listening to a melody interact with the observer's execution of piano play. This interaction is thought to occur because the execution of musical-action and the perception of both musical-action and musical-sound share a common representation in which the frontoparietal network is involved. However, it is unclear whether the perceptions of observed piano play and listened musical sound use a common neural resource. The present study used near-infrared spectroscopy to determine whether the interaction between the perception of musical-action and musical-sound sequences appear in the left prefrontal area. Measurements were obtained while participants watched videos that featured hands playing familiar melodies on a piano keyboard. Hand movements were paired with either a congruent or an incongruent melody. Two groups of participants (nine well-trained and nine less-trained) were instructed to identify the melody according to hand movements and to ignore the accompanying auditory track. Increased cortical activation was detected in the well-trained participants when hand movements were paired with incongruent melodies. Therefore, an interference effect was detected regarding the processing of action and sound sequences, indicating that musical-action sequences may be perceived with a representation that is also used for the perception of musical-sound sequences. However, in less-trained participants, such a contrast was not detected between conditions despite both groups featuring comparable key-touch reading abilities. Therefore, the current results imply that the left prefrontal area is involved in translating temporally structured sequences between domains. Additionally, expertise may be a crucial factor underlying this translation.

## Introduction

Playing a musical instrument, such as the piano, requires coordinating hand movement sequences within a strictly defined temporal structure. The interaction between sensory and motor systems is beneficial because the sound produced by each action enables the performer to compare his or her ongoing performance with the performance that is anticipated.

Behavioral study revealed that when auditory feedback is experimentally altered and when a “wrong” sound precedes the action, motor performance is disrupted in musicians but not in musically naïve controls (Drost et al., 2005a,b; Pfordresher, 2006; Furuya and Soechting, 2010). Additionally, Novembre and Keller (2011) instructed musicians to watch silent videos of a hand playing 5-chord sequences and to imitate the sequences. When the observed hand played a chord that was harmonically incongruent with the preceding musical context, the imitation was disturbed. These studies indicate that the perception of sound and observation of action are likely not independent from the execution of behavior.

Brain imaging studies using functional Magnetic Resonance Imaging (fMRI) have demonstrated a neural correlate for an auditory-motor interaction. For instance, Lahav et al. (2007) found significant responses in the bilateral frontoparietal motor-related network (including Broca's area and its right homolog, the premotor region, the intraparietal sulcus, and the inferior parietal region) of non-musicians when they listened to melodies that they had practiced but not when they listened to untrained melodies (also see Mutschler et al., 2007). Furthermore, Bangert et al. (2006) examined the auditory-motor interaction that is related to musicianship. Pianists showed increased activity compared to non-musicians in a distributed cortical network while listening to piano melodies and while playing a muted keyboard. A conjunction analysis revealed core brain regions that activated when the pianist listened to melodies and when they played them silently. These regions were the left motor-related regions, including Broca's area and supplementary motor and premotor areas, as well as the bilateral superior temporal gyrus and supramarginal gyrus (also see Baumann et al., 2007).

In addition to an auditory-motor interaction, a visual-motor interaction can also occur because particular sounds are assigned to specific locations on a piano keyboard. Studies revealed visual-motor co-activations. For instance, the observation of a silent hand action of piano playing compared with the observation of musically irrelevant hand movements induced stronger differences in activations of the frontoparietal motor-related regions (Broca's area, the bilateral ventral and dorsal premotor region, and the bilateral intraparietal sulcus) along with the superior temporal area in musicians than in musically naive controls (Haslinger et al., 2005; also see Hasegawa et al., 2004). Additionally, in musically naive participants who were trained to play the piano, fMRI revealed that the observation of finger movements corresponding to the audio-motor trained melodies was associated with a stronger activation in the cluster of the left rolandic operculum extending into Broca's area than the observation of hand movements corresponding to untrained sequences (Engel et al., 2012).

Taken together, these studies showed that (1) the silent execution of piano performance, (2) the observation of silent finger movement on a keyboard and (3) listening to sound sequences typically elicited the activation of similar left frontoparietal motor-related regions. Further, such co-activation is more prominent in musicians than non-musicians and during listening to or watching trained compared with untrained melodies or performance. These findings indicate that such an interaction may be established by a long-term association between a particular body movement and a particular sound or key.

In the musical domain, the inferior frontal region is generally responsive bilaterally in the auditory perception of harmony violation (Maess et al., 2001; Koelsch et al., 2002; Tillmann et al., 2003) and in melody and harmony discrimination (Brown and Martinez, 2007). Brown et al. (2013) discovered the core regions for the playing of musically structured sequences; these include a slightly left-lateralized inferior frontal area (cf. Bengtsson and Ullén, 2006).

In the action domain, the left inferior frontal cortex contributes to the imitation of hand movement (Iacoboni et al., 1999), to action understanding (Cross et al., 2006; Pobric and Hamilton, 2006; Wakita and Hiraishi, 2011) and to the imagery and execution of tool use (Higuchi et al., 2009). This region features the hierarchical and sequential processing of observation and the execution of action (cf. Koechlin and Jubault, 2006; Clerget et al., 2009, 2012, 2013; Wakita, 2014).

Taken together, the left inferior frontal area may be involved in diverse tasks requiring sequence processing. Notably, this brain region is also involved in analyzing the sequential organization of visual symbols (Bahlmann et al., 2009; Alamia et al., 2016). Therefore, the auditory-motor and visual-motor interactions noted above are assumed to occur because both the perception and execution of music involve the analysis and organization of musical temporal structures.

Whether the perceptual interactions between the observation of the action of keyboard playing (i.e., musical-action sequence) and the listening of the sounds of melody (i.e., musical-sound sequence) also occur in the inferior frontal area remains unknown. Given the contribution of the left inferior frontal area to diverse tasks requiring sequence processing, the perceptual interaction between musical-action, and musical-sound may appear in the left inferior frontal area.

Thus far, several tasks have been adopted to study the brain activity involved in observing musical-action. The task in Novembre and Keller (2011) and Sammler et al. (2013) successfully revealed the participants' structural processing ability of perception and planning of action by presenting chord sequences silently played by another person's hand that ended either with a harmonically congruent or incongruent chord. However, sophisticated knowledge of chord progression was required for the task; thus, less-trained participants cannot perform the task. Additionally, in the task of Hasegawa et al. (2004), participants watched a muted hand playing piano pieces that the participants had to identify by name. As the authors implied, musical action consists of hierarchical structures in that the visual processing of structured key-press sequences and the integration of this information with the location of the fingers on the piano keyboard are essential to correctly identify harmony or melody via the observation of silent hand motion. In their study, even non-musicians could identify some of the stimulus pieces; however, behavioral performance was generally low. Thus, the degree to which brain activity (particularly in the less-trained participants) reflected the sequential analysis of the observed action was unclear. Therefore, I created stimuli from familiar musical pieces that all participants could identify by name when they observed the pieces being played silently via key-touching movements.

The present study tested the effects of the interaction between the perception of musical-action and musical-sound sequences within the left prefrontal area. Well-trained and less-trained participants watched videos that featured the right hand playing familiar melodies. Each hand movement was paired with task-irrelevant sounds that were either congruent or incongruent with the observed “melodies.” The activation of the left inferior frontal area was measured using near-infrared spectroscopy (NIRS). The NIRS results for congruent and incongruent conditions were compared to reveal the involvement of the left prefrontal area in the perceptual interaction between sequences of musical-action and musical-sound. If this brain region demonstrated greater activation under incongruent compared with congruent conditions, the incongruent musical-sound sequences were deduced to interfere with the analysis of musical-action sequences. Thus, the left prefrontal area may be involved in the perception of sequences regardless of whether they are composed of sound or action. Additionally, if the NIRS results were correlated with the duration of piano training, motor experience would be a critical factor affecting the sequential structures represented in the left prefrontal area.

## Methods

### Participants

Eighteen healthy, right-handed adults aged 23–40 years were recruited for this study. Handedness scores were determined using the Edinburgh Handedness Inventory (Oldfield, 1971). Participants in the well-trained group (females, 9; mean age, 29.2 years; range, 23–34 years) had received formal piano lessons for at least 8 years (mean, 11.2 years; range, 8–16 years). Participants in the less-trained group (males, 2, females, 7; mean age, 28.6 years; range, 24–40 years) had received formal piano lessons for <8 years (mean, 3.7 years; range, 1–7 years). All participants were able to identify the stimulus melodies by watching the hand movements in each video. Prior to the start of the experiment, all participants were informed about the nature of the experimental procedures and provided written informed consent. This study was approved by the Human Research Ethics Committee of the Primate Research Institute, Kyoto University.

In the present experiments, the well-trained and less-trained groups were not balanced regarding participant sex. Notably, no males were included in the well-trained group. One may suspect that such a distribution pattern influenced the results. However, in the less-trained group, the NIRS results of the male participants were within the range of those of the female participants (Supplementary Figure 1). Therefore, the biased distribution of participant sex likely did not influence the results. Thus, I did not separately assess the data from the female and male participants.

### Stimulus

**Figure 1:**
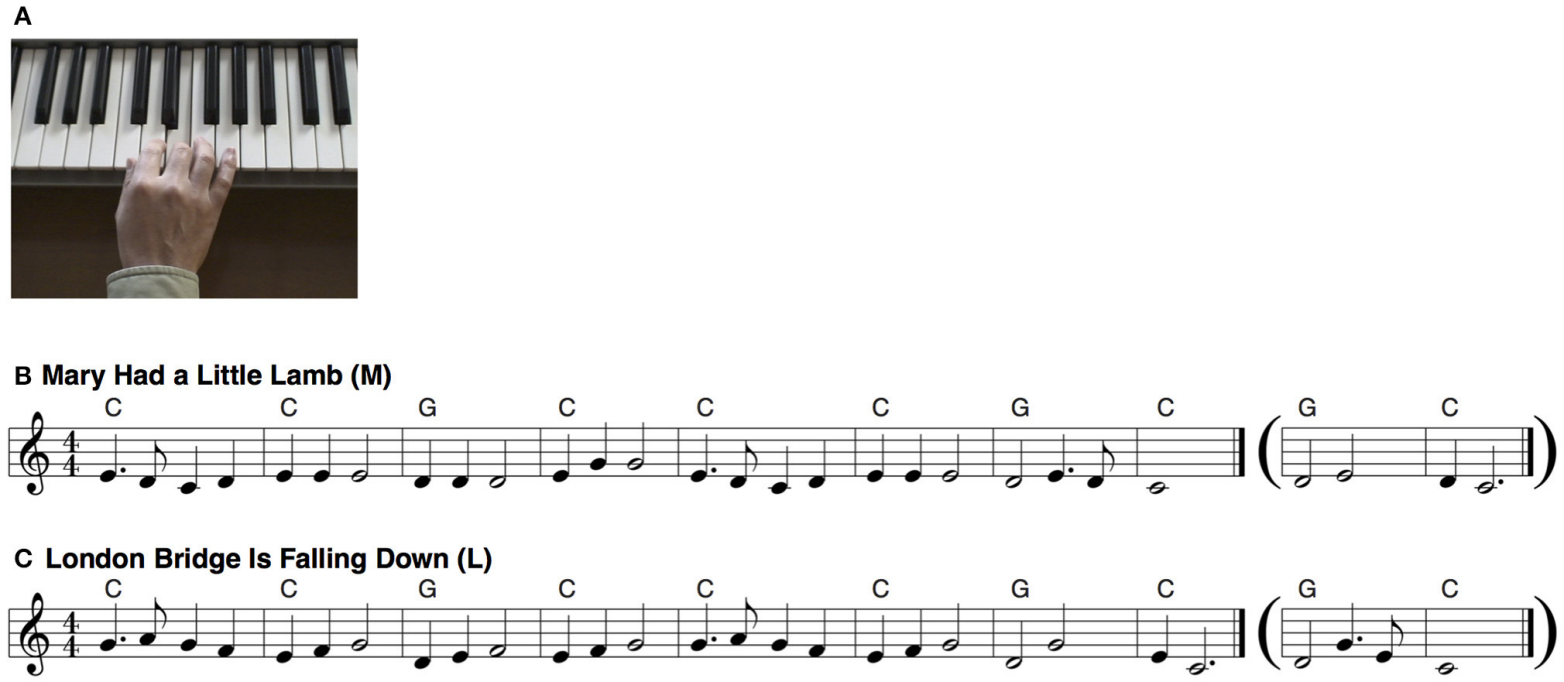
**Stimuli**. Static example of a stimulus video (A). Scores of melodies of “Mary Had a Little Lamb” **(B)** and “London Bridge Is Falling Down” **(C)**. In the congruent condition, either a musical-action sequence featuring melody M or L was presented with a task-irrelevant musical-sound sequence of M or L, respectively (Supplementary Video 1). In the incongruent condition, either a musical-action sequence featuring melody M or L was presented with a task-irrelevant musical-sound sequence of L or M, respectively, in which hand movements were modified to match the key-touch timing and sound onset timing. Accordingly, in the last two bars of incongruent videos, the observed hand was playing the melody shown in the parentheses (Supplementary Video 2).

For stimulus videos, hand movements playing “Mary Had a Little Lamb” (M) and “London Bridge Is Falling Down” (L) (key, C major; duration, 8 s) on the keyboard were captured, and piano sounds were recorded (640 × 480 pixels and 16-bit/44.1-kHz resolution). Next, two types of stimulus videos were generated, featuring congruent, and incongruent stimuli. Congruent stimuli were created by combining hand movements and sound sequences from the same melody (e.g., the participants observed the hand movements playing M and heard melody M). In contrast, incongruent stimuli were created by combining hand movements and sound sequences from different melodies (e.g., the participants observed the hand movements playing M but heard melody L). Consequently, two congruent videos and two incongruent videos were generated (Figure 1 and Supplementary Video 1). The order of congruent and incongruent trials was randomized for each participant. Key-touch timing was the same between the original M and L melodies, with the exception of the last two bars. Accordingly, to maintain the simultaneity of key-touch timing and sound onset timing in the incongruent condition, the hand played the modified melodies in the last two bars (Figure 1, parentheses) such that the key-touch timing matched the sound onset timing. In the post-experiment debriefing, no participants reported unnatural hand movements despite these modifications. Each 8-s video was repeated twice to generate a 16-s stimulus video. Fade-in and fade-out periods (1 s) were inserted between the videos to prevent participants from using the initial hand position and tone to judge the melody. Stimulus videos were presented on a 17-inch liquid crystal display monitor placed ~70 cm from the participants' heads. Stimulus sounds were delivered via a loudspeaker.

## Procedure

### Action observation task

Each experimental session consisted of 12 trials. Each trial lasted 16 s, during which one of the four stimulus videos was presented. All four videos were presented three times in a pseudorandom order, with each session starting with a congruent stimulus such that the participants could start the experiment confidently. Each stimulus was presented once over four successive trials, and the same melody (M and L in either action or sound aspects) and/or congruency (congruent and incongruent) condition was repeated in no more than three consecutive trials. The length of the inter-trial interval was 25 s.

The participants were instructed to watch the stimulus videos carefully to identify the “melody” performed by the hand while ignoring the auditory sequence. Participants were not required to present their judgment during the task session to avoid the possible influence of body movement on the NIRS results. The beginning of each stimulus video was accompanied by a beep. Participants were instructed to open their eyes gently when they heard the tone and close their eyes when the stimulus video ended. Therefore, the participants' eyes were closed during the rest period. The entire experiment lasted ~30 min.

Prior to the start of the recording session, the participants underwent eight practice trials (four stimulus videos × two trials) with the experimental stimuli to confirm that they understood the instructions. Consequently, all the participants perfectly identified the “melodies” performed by the hand without being disturbed by the auditory sounds under both congruent and incongruent conditions. The participants therefore understood that musical-action sequences were task-relevant but that musical-sound sequences were task-irrelevant.

### Near-infrared spectroscopy measurements

Cortical activity was continuously recorded throughout the experiment, wherein relative changes in oxy-hemoglobin (oxy-Hb) concentrations were measured using an NIRS system (ETG-100, Hitachi Medical Corporation, Tokyo, Japan). Strong correlation was reported between oxy-Hb change and fMRI signal (Strangman et al., 2002). The sampling rate was set to 10 Hz. Sixteen optodes in a 4 × 4 lattice pattern forming 24 channels were positioned on the left hemisphere. Notably, the emitter-detector distance was fixed at 3 cm despite the variation in the head size of the participants, and it was difficult to place optodes in a similar angle and position across participants; thus, one recording channel may have corresponded to different positions on the head. Therefore, a single recording channel was determined as the point between the optode nearest F7 on the international 10–20 system and its posteriorly adjacent optode. The F7 placement site is reported to project onto the cortical surface of the anterior portion of the inferior frontal cortex [Brodmann area (BA) 45/47] (Okamoto et al., 2004, 2006; Koessler et al., 2009); thus, the NIRS results obtained were expected to reflect activity in the left inferior frontal area, which was posterior to BA 45/47. Notably, there is substantial variation in the precise location and topographic extent of the left inferior frontal area between individuals (Amunts et al., 1999). It may be difficult to ensure the contribution of different subdivisions within the inferior frontal area to the NIRS signal. However, the NIRS data can be safely taken to be obtained from the left prefrontal area in this study.

The cortical responses for each trial were stimulus-locked and extracted from the raw oxy-Hb time series data. Pulsatile fluctuations were removed by smoothing the oxy-Hb time series backward in time using a 5-s moving window. Baseline drift was corrected using linear interpolation between the time point of stimulus onset and that of the next trial. The oxy-Hb time series data from congruent and incongruent trials were individually averaged independently of the melody type. Finally, the mean oxy-Hb was calculated per time point within a peri-stimulus period [between 5 and 21 s after stimulus onset, accounting for a delay of the blood-oxygen-level-dependent (BOLD) responses of 5 s] for each condition. The averaged oxy-Hb values were then analyzed using a mixed-design analysis of variance (ANOVA) [in which the stimulus condition (congruent vs. incongruent) was a within-subject factor and the degree of expertise (well-trained vs. less-trained) was a between-subject factor] to assess the interference of music on the activation of the left prefrontal area during action observation. Significance was set at 0.05.

Further, the degree of sensitivity of the left prefrontal area to the interaction between musical-action and musical-sound sequences may be influenced by the experience. Therefore, the relationship between such sensitivity and the duration of piano training was assessed. First, subtracting the averaged oxy-Hb value in the congruent condition from that in the incongruent condition was individually calculated as an index that shows the sensitivity to the interaction. Then, the correlation between such contrast and the duration of piano training was evaluated using Spearman's rank correlation test. Significance was set at 0.05.

## Results

**Figure 2:**
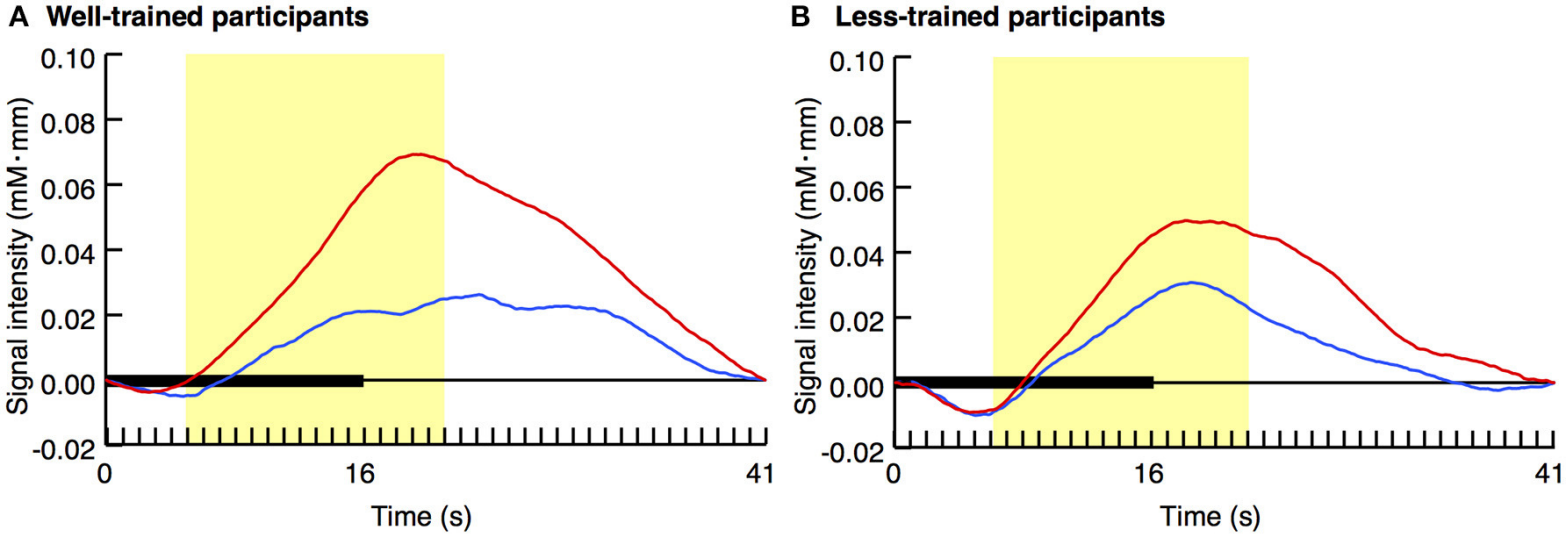
**Average time series data of changes in oxy-Hb concentration after stimulus onset**. The results of the congruent (blue lines) and incongruent (red lines) trials for well-trained **(A)** and less-trained **(B)** participants are shown. Horizontal thick bars indicate the trial period, in which an 8-s video was repeated twice (i.e., stimulation period). This period was followed by a 25-s inter-trial interval. The yellow areas indicate the peri-stimulation period. Data included in this period (160 data points) were individually averaged for the later analysis.

Figure 2 displays the average activity in the left prefrontal area over time with regard to both the congruent and incongruent conditions. In the well-trained group (A), a difference in the pattern of signal change was evident between the two conditions. However, such a contrast was not observed in the less-trained group (B).

**Figure 3:**
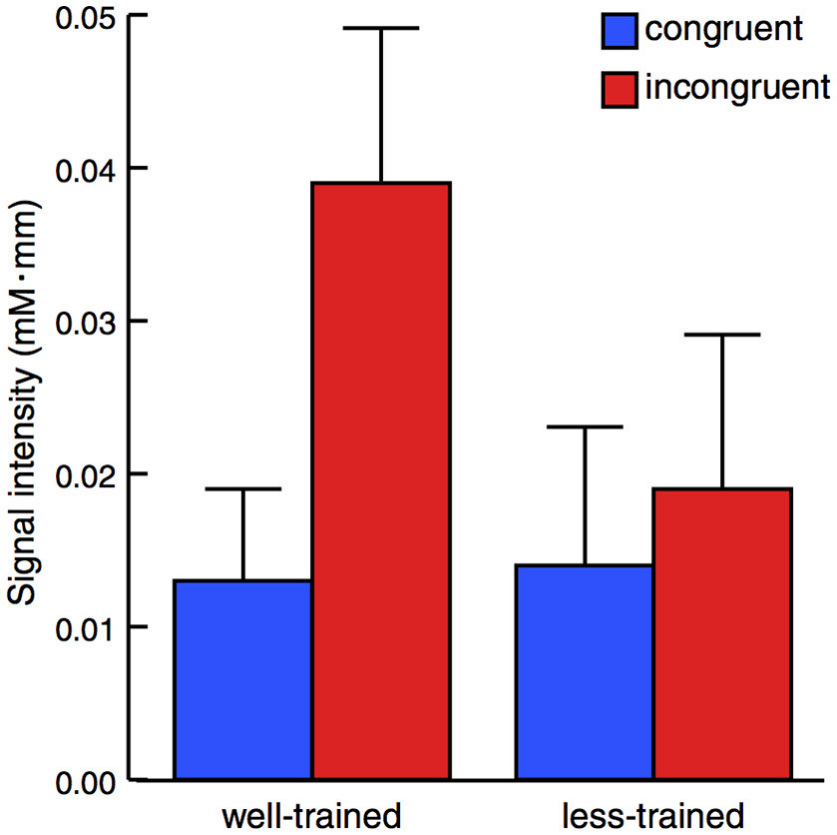
**Average changes in oxy-Hb concentration within the peri-stimulation period**. The results of the congruent (blue) and incongruent trials (red) were plotted for well-trained (left) and less-trained participants (right). The plots indicate the mean + 1 *SE*. Mixed-design ANOVA demonstrated a statistically significant main effect between congruent and incongruent conditions but not between well-trained and less-trained participants with a significant effect of interaction.

The activation of the left prefrontal area during the peri-stimulus period (mean ± *SE*) is displayed in Figure 3. A mixed-design ANOVA demonstrated a statistically significant main effect between the congruent and incongruent conditions [*F*_(1, 16)_ = 13.38, *p* = 0.002] but not between the well-trained and less-trained participants [*F*_(1, 16)_ = 0.703, *p* = 0.414]. Additionally, a statistically significant effect for interaction [*F*_(1, 16)_ = 7.056, *p* = 0.0172] was revealed. These results show that activation in the left prefrontal area was remarkably high in the well-trained participants under the incongruent condition.

**Figure 4:**
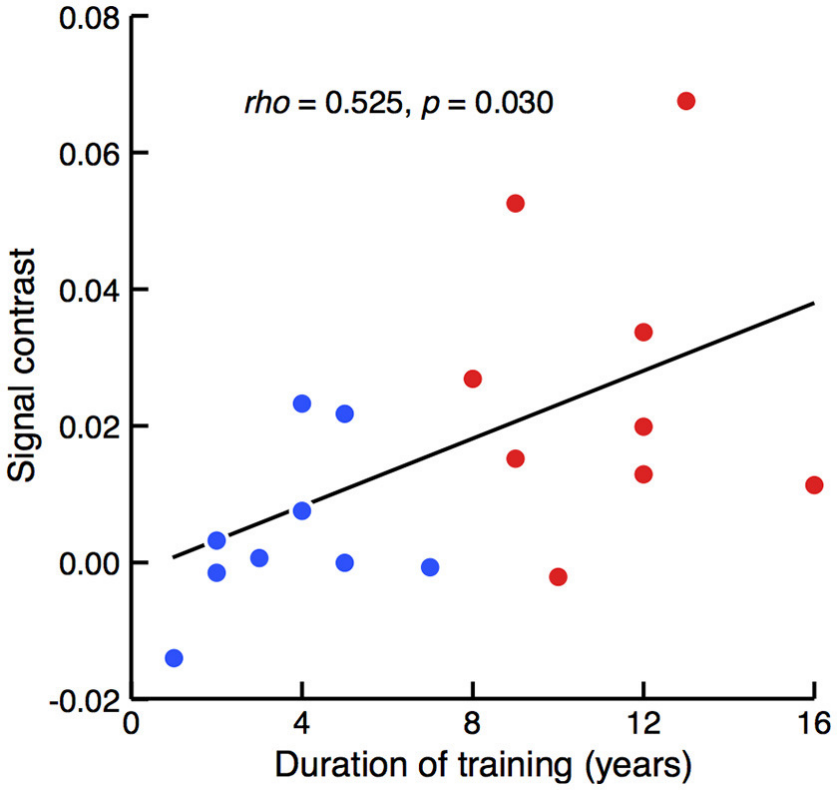
**Correlation between signal contrast of congruent and incongruent conditions and the duration of piano training**. Red and blue dots represent the results of well-trained and less-trained participants, respectively. Spearman's rank correlation test revealed that between-condition differences of NIRS signals were significantly larger in the participants with a longer duration of piano training.

The relationship between the degree of sensitivity to such involvement of the left prefrontal area and the duration of piano training was then assessed. A correlation analysis of the entire population revealed that the between-condition difference in mean signal intensity correlated with the duration of piano training [Figure 4; Spearman's rank correlation test, *rho*_(16)_ = 0.525, *z* = 2.166, *p* = 0.030]. Therefore, activation in the left prefrontal area in response to cross-domain interference became evident in participants with a long history of piano training.

## Discussion

### Interaction between perception of action and sound sequences

The aim of the present study was to determine whether an interaction between the perception of the sequential organization of musical-action and musical-sound appears in the left prefrontal area. Enhanced NIRS signals were detected in the left prefrontal area under an incongruent condition relative to a congruent condition, indicating a higher processing load when the perception of musical-action sequences was subject to interference from task-irrelevant musical-sound sequences. Notably, this interference effect was evident in the well-trained group.

However, the participants were asked to identify melodies by observing silent musical-action; thus, the present NIRS results could be caused by unexpected local features of the hand movements. For example, there were non-standard key-touching hand movements in the last two bars of incongruent stimuli occurring at the 6 and 14 s points for a 2-s duration (although no participants reported that the hand movements were unnatural). This condition-unique feature could have caused a between-condition contrast in NIRS signal in the well-trained participants. When considering the typical delay in BOLD response after stimulation, however, the NIRS change in the well-trained participants in the incongruent condition appeared to occur soon after stimulus onset and earlier than that in the congruent condition. Therefore, it is difficult to explain the NIRS contrast in the well-trained participants solely according to their detection of the unexpected action.

Additionally, there were stimulus-unique sequences of finger movements in the melody M, in which the same key was depressed three times, i.e., [EEE], [DDD], and [EEE]. If melody identification by less-trained participants was dependent on such local cues, their between-condition contrast in NIRS signals could have been reduced. However, although precise response latencies were not available (I failed to measure the response latency to identify the melodies), all participants identified the melodies perfectly and with equal speed in the practice trials regardless of whether the musical-action sequences contained this feature. Moreover, because the participants watched the stimulus videos only eight times, including four incongruent videos until the NIRS measurement began, it is difficult to speculate that they formulated an efficient strategy to identify the melody. Taken together, the NIRS results likely represented an interaction between the perception of musical-action and musical-sound sequences. However, data were obtained from only one selected channel; thus, it is difficult to estimate how specific the interaction effect was to the recorded region.

### Degree of expertise and activity in the left prefrontal area

As the correlation analysis indicated, the length of training history may be a crucial factor affecting how the left prefrontal area represents the interaction between the perception of musical-action and musical-sound sequences. Drost et al. (2005a,b) and Stewart et al. (2013) proposed that automatic sound-to-key association (i.e., anticipatory motor planning) increased with experience. In the current experiment, therefore, incoming congruent sound sequences may have enabled the well-trained participants to anticipate how observed hand movements should be performed and to then efficiently analyze the hand movement sequences. However, under an incongruent condition, in which the touched keys were always associated with conflicting sounds, the processing of hand movements sequences likely required controlled cognitive processes. Consequently, the increased NIRS signal conceivably represented an elevated cognitive demand in analyzing the observed action sequences.

Sound-to-key associations in less-trained participants may not occur as automatically as in well-trained participants despite the former group's demonstrated touch reading ability. Even sound sequences that were congruent with the observed action sequences could not strongly facilitate the processing of hand movement sequences. Therefore, an equal level of cognitive control was likely required to form an association between the sound and the key regardless of whether hand movement sequences were paired with either congruent or incongruent sound sequences. Accordingly, a task-dependent brain activation may not be found in the left prefrontal area. However, our NIRS apparatus quantifies only relative hemoglobin concentration changes using the start point of measurement as the reference. The absolute baseline value was unknown; therefore, whether the similar levels of NIRS signals in the less-trained participants in both conditions were due to equally high or equally low brain activation could not be determined.

I used only familiar musical pieces, and all participants could identify the pieces solely by observing silent hand movements on a keyboard. Accordingly, the potential differences in musical expertise between participants were unknown. Drost et al. (2005a,b) reported that cognitive interference was detectable in response latency but not behavioral performance. Therefore, if response latency were assessed during the practice trials, a prolonged latency in the congruent condition may have reflected the participants' sensitivity to cognitive interference. Additionally, the identification ratio in response to key-touching movements playing many musical pieces with various levels of popularity [as adopted in Hasegawa et al. (2004)] would also be useful to evaluate the musical skill of individual participants. Future studies using such behavioral scores may more precisely (than training experience) explain the influence of the degree of musical expertise and cognitive interference on the brain activity that represents the interaction between the perception of musical-action and musical-sound sequences.

### Interaction between action and sound sequences in the left prefrontal area

An interference paradigm was adopted to study the interaction between the perception of musical-action and musical-sound sequences. Enhanced brain activation under incongruent conditions implies that musical-action and musical-sound sequence perceptions may have competed in a common processor in the left prefrontal area. Notably, the current task required the transformation of a sequence of individually meaningless key-pressing movements by integrating such information with the location of the fingers on the piano keyboard and the duration of key pressing into a temporally structured and meaningful melody. Incongruent sound sequences may have added conflicting information to this temporal processing; enhanced activation in the left prefrontal area was surely related to such temporal computation.

There is a posterior-anterior functional gradient of the prefrontal area depending on the level of the temporal organization of action (Koechlin and Jubault, 2006; for reviews, see Koechlin and Summerfield, 2007 and Jeon, 2014): the activation of BA 6, BA 44, and BA 45 are involved in action planning of single acts, simple chunks, and superordinate chunks, respectively (Koechlin and Jubault, 2006). Thus, despite a difference between the planning and perception of action, if an enhancement of activation is found in BA 6 or BA 44, for instance, action perception is assumed to experience interference from the sound sequence at the level of processing individual finger movements, such as E-D-C-D-E-E-E (in the case of melody M), or at the level of processing simple chunks, such as [EDCD]-[EEE]. However, in the present study, brain activity was measured using NIRS, which features a lower spatial resolution than fMRI and does not permit the precise identification of task-specific sub-regions. Future fMRI studies revealing where such interference is represented in the brain regions would also clarify the level of temporal processing that is the origin of the interaction between the perception of musical-action and musical-sound sequences.

### Role of the prefrontal area in interference suppression

Thus far, I interpreted the results from the viewpoint of shared representation between musical-action and musical-sound perception. However, one alternative explanation may be that an elevated activation of the left prefrontal area may reflect an increased cognitive control related to a conflict-resolution processes or interference suppression. Neuroimaging studies have revealed a role for the left inferior frontal cortex in overcoming proactive interference from preceding items in working memory (for a review, see Jonides and Nee, 2006). Similarly, activation of the right inferior frontal cortex during interference resolution has been linked to response inhibition (for a review, see Aron, 2011) or reprogramming motor plan (Mars et al., 2007) in response to external signals. Accordingly, in well-trained participants with a strong key-to-sound association, a violation in key-to-sound relation may be sufficient to interfere with maintaining sequences of key-touching movements in a working memory store during melody perception. In less-trained participants, such violation may not influence their melody perception because their key-to-sound association would be less consolidated. Therefore, the elevated NIRS signal in the present study may be explained by interference suppression without assuming an interaction between the representations of musical-action and musical-sound.

To confirm the mechanism underlying an elevated activation of the left prefrontal area, future studies should introduce incongruent stimuli, in which sequences of hand movements are paired with sequences of sounds that do not evoke a musical representation and the participants cannot abstract chunks from the sound sequences. If such incongruent stimuli enhance the activation of the left prefrontal area during the observation of hand movements sequences compared with congruent stimuli, enhanced activation of this brain area in the present study can be considered to reflect an interference suppression rather than an interaction between musical-action and musical-sound sequences.

## Conclusion

The present study demonstrated that well-trained participants exhibited enhanced activation of the left prefrontal area when watching musical-action sequences paired with incongruent vs. congruent musical-sound sequences. Less-trained participants, who had touch-reading ability, did not show such a between-condition contrast, indicating that long motor training is necessary for the left prefrontal area to become sensitized to action-sound interactions. Although it was difficult to determine functional specificity to the recorded area, perceptual interaction between musical-action and musical-sound sequences was suggested in the left prefrontal area. Future studies could more precisely investigate which subdivision of the prefrontal area is responsible for perceiving interactions between musical-action and musical-sound sequences.

## Author contributions

MW designed and conducted experiments, analyzed results, and wrote the paper.

### Conflict of interest statement

The author declares that the research was conducted in the absence of any commercial or financial relationships that could be construed as a potential conflict of interest.

## Supplementary material

Supplementary Figure 1 **Comparison of results between less-trained male and female participants. (A)** Comparison of NIRS results of identical participants between congruent and incongruent conditions. Points from the same individual are connected. **(B)** Correlation between signal contrast of congruent and incongruent conditions and the duration of piano training. This is the same plot as shown in Figure 4. Red open circles represent the results of the well-trained female participants. Blue open and filled circles represent the results of the less-trained female and male participants. In the less-trained group, the NIRS results of the male participants were within the range of those of the female participants.

Supplementary Video 1 **Example of a congruent stimulus**. In this video, both the melody played by the hand and the auditory sequence paired are “Mary Had a Little Lamb.”

Supplementary Video 2 **Example of an incongruent stimulus**. In this video, the melody played by the hand is “Mary Had a Little Lamb;” however, the auditory sequence paired is “London Bridge Is Falling Down.”
