# Supplementary material for: Interaction between Perceived Action and Music Sequences in the Left Prefrontal Area
Source: Front Hum Neurosci. 2016 Dec 27;10:656. doi: 10.3389/fnhum.2016.00656 (PMC5186772; doi:10.3389/fnhum.2016.00656)
Supplement: Supplementary Figure 1 — Comparison of results between less-trained male and female participants. (A) Comparison of NIRS results of identical participants between congruent and incongruent conditions. Points from the same individual are connected. (B) Correlation between signal contrast of congruent and incongruent conditions and the duration of piano training. This is the same plot as shown in Figure 4. Red open circles represent the results of the well-trained female participants. Blue open and filled circles represent the results of the less-trained female and male participants. In the less-trained group, the NIRS results of the male participants were within the range of those of the female participants. [file Presentation1.PPTX]

## Slide 1
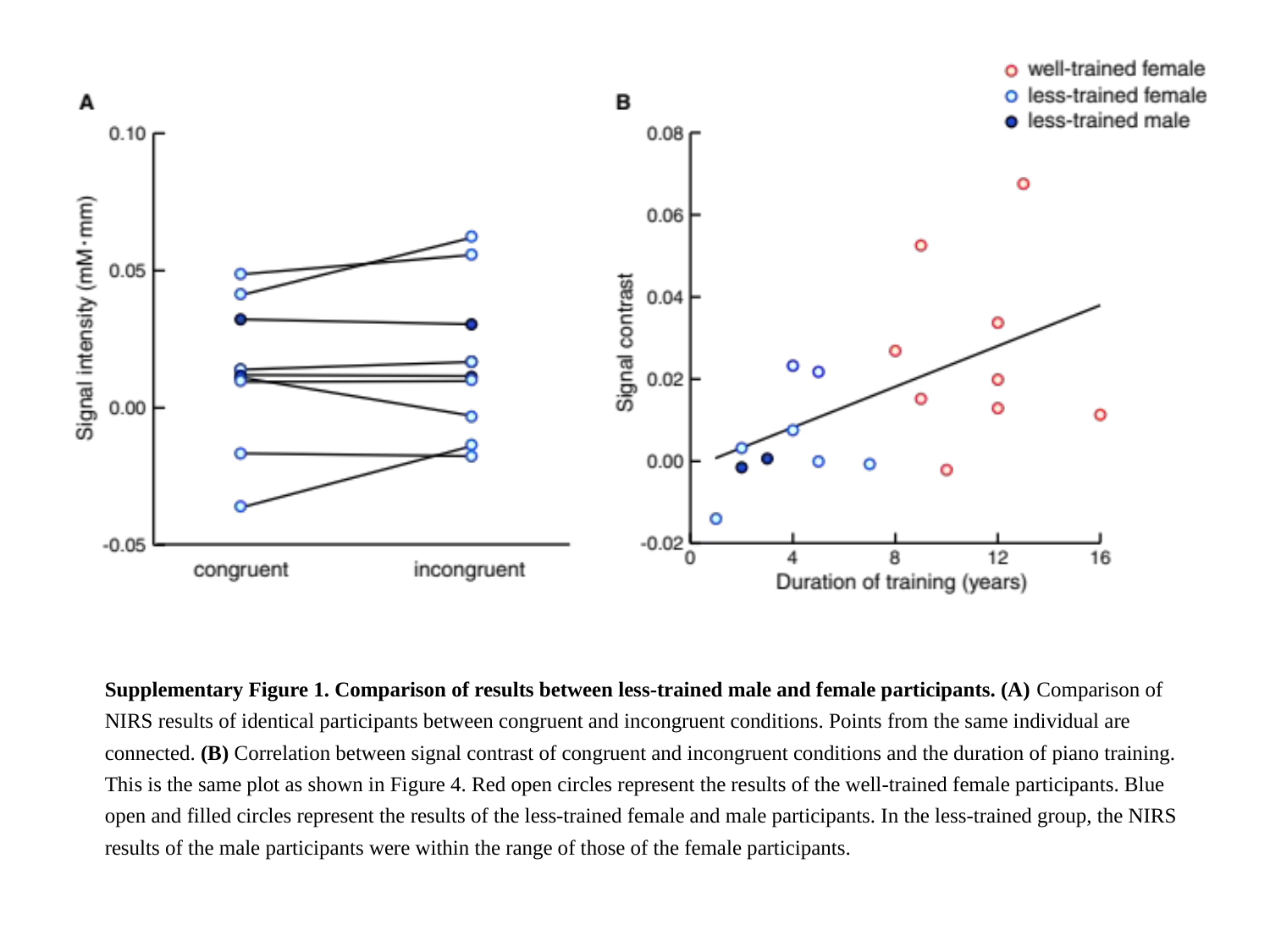

Supplementary Figure 1. Comparison of results between less-trained male and female participants. (A) Comparison of NIRS results of identical participants between congruent and incongruent conditions. Points from the same individual are connected. (B) Correlation between signal contrast of congruent and incongruent conditions and the duration of piano training. This is the same plot as shown in Figure 4. Red open circles represent the results of the well-trained female participants. Blue open and filled circles represent the results of the less-trained female and male participants. In the less-trained group, the NIRS results of the male participants were within the range of those of the female participants.
